# Locked Central Fracture Dislocation of the Hip in a Child after Low-Energy Trauma

**DOI:** 10.1155/2017/6873484

**Published:** 2017-11-27

**Authors:** Alexandre H. Nehme, Jack C. Daoud, Hicham G. Abdelnour, Jad N. Bou Mounsef, Ramzi C. Moucharafieh, Joseph W. Wehbe

**Affiliations:** ^1^Department of Orthopedic Surgery and Traumatology, Saint Georges University Medical Center, Balamand University, P.O. Box 166378, Achrafieh, Beirut 1100 2807, Lebanon; ^2^Orthopedic Surgery Department, University of Illinois at Chicago, Chicago, IL, USA

## Abstract

We present the case of a 13-year-old boy who sustained a locked central fracture dislocation of the right acetabulum following a bicycle fall. Immediate external reduction maneuvers under general anesthesia were unsuccessful due to intrapelvic entrapment of the femoral head. Open reduction internal fixation was achieved 48 hours later. After an initial satisfactory postoperative course, the patient ended up developing severe hip osteoarthritis 16 months after the procedure. The rarity of this injury in children is discussed, with its possible implications on joint congruity and potential growth injury.

## 1. Introduction

Fractures and dislocations of the pelvis and proximal femur in children are rare injuries and usually the result of high-energy trauma [1–7]. They account for approximately 2% to 5% of annual level I trauma center admissions [2]. Acetabular fractures form 1% to 15% of these cases [1]. Low-energy trauma acetabular fractures are even less common in children, and very scarce reports exist in the English literature describing this entity. Nodzo et al. described the case of a 15-year-old hockey player who sustained a nondisplaced bilateral anterior column and wall acetabular fracture after low-energy trauma that was managed conservatively. The patient had complete resolution of his symptoms by 10 weeks [1].

We report the case of a 13-year-old boy who sustained a locked intrapelvic dislocation after falling off his bike. The aim of this case report is to review the pathophysiology and mechanism of this entity and discuss our method of treatment, result, and our recommendations for treating similar cases.

## 2. Case Report

A previously healthy 13-year-old boy sustained a fall when riding his bike slowly on a Rocky Mountain road. Immediately after the fall, he was unable to walk, and his right lower limb was locked in internal rotation. The impact was directly at the region of his right hip trochanter. He was transferred quickly to the nearest emergency room of a level III hospital. On presentation, the patient had his entire vital signs stable and his right lower limb fixed in adduction, internal rotation. His right lower limb was neurovascularly intact.

**Figure 1:**
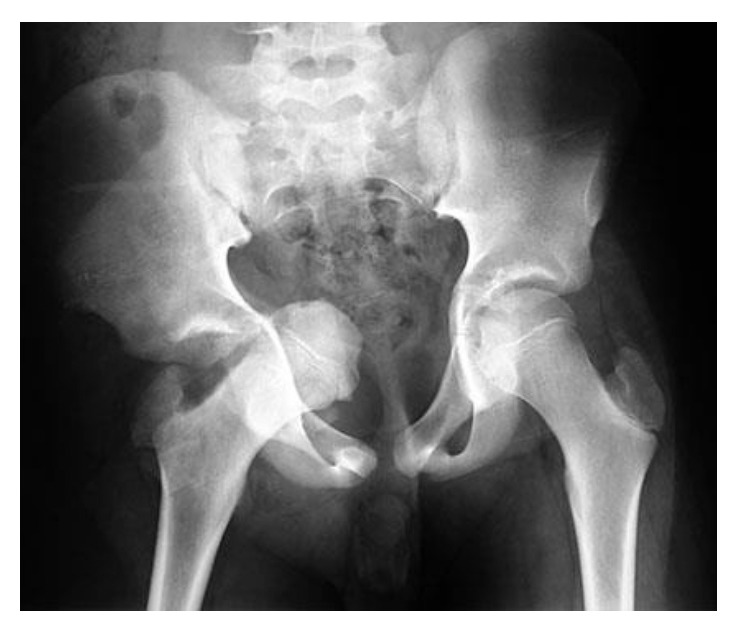
Displaced right acetabular fracture dislocation with anterior column fracture associated with a central migration of the right femoral head.

Initial radiographic assessment revealed that he had sustained a displaced right acetabular fracture dislocation with anterior column fracture associated with a central migration of the right femoral head through the quadrilateral surface and an inferiorly and posteriorly displaced Salter II-III greater trochanteric fracture (Figure 1).

**Figure 2:**
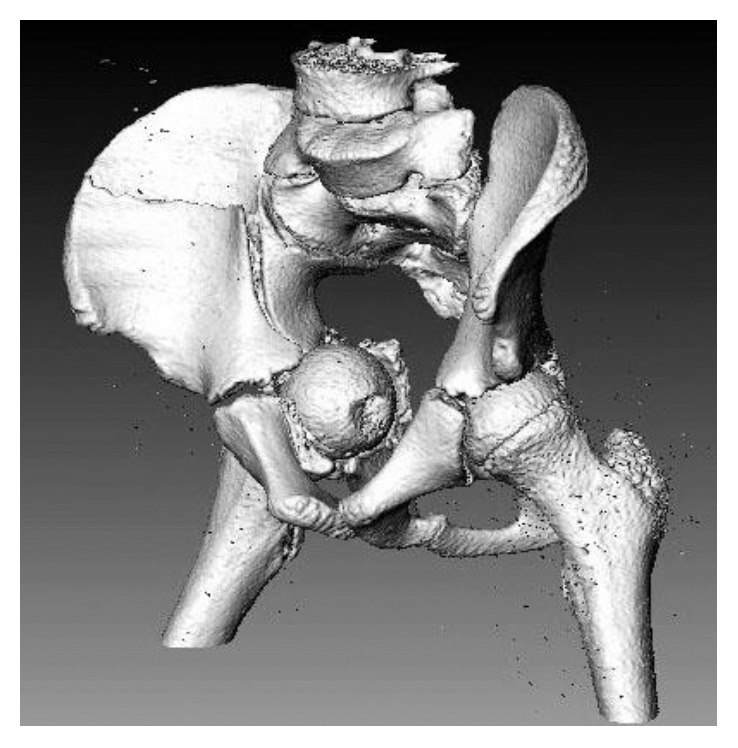
3D reconstruction showing the medial dislocation with entrapment of the femoral head.

Computed tomography scan with 3D reconstruction imaging was obtained and confirmed the injury pattern. It showed that the femoral head-neck junction was entrapped through the hiatus created by the medially displaced quadrilateral surface between the anterior and posterior columns (Figure 2).

He was immediately brought to the operating theater where a closed reduction under general anesthesia and complete muscle relaxation was carried. After 30 minutes of unsuccessful attempts of closed reduction, the patient was returned to his room afterwards for monitoring and transferred 48 hours after his initial admission to our level I trauma center. After informing the parents about the severity of the situation with the possibility of avascular necrosis of the femoral head (AVN) due to delay in reduction, the patient was transferred to the operating room, where a classic ilioinguinal approach was done [8, 9]. After superficial and deep dissection, we used only the lateral (lateral to iliopsoas) and middle (between iliac vessels and iliopsoas) windows of this approach, thus accessing the iliac wing, the pelvic brim, the quadrilateral surface, and portion of the superior pubic ramus.

A lateral incision was done over the greater trochanter region where a corkscrew was introduced through the lateral femoral cortex distal to the trochanteric physis in order to perform a lateral traction to disengage the femoral head from the inner pelvis. In spite of multiple trials, the femoral head was stuck, and the reduction was not successful until we used a cob elevator as a lever arm placed in the anterior column fracture to exaggerate the displacement, thus increasing the anteroposterior diameter of the acetabulum and allowing the reduction of the femoral head.

**Figure 3:**
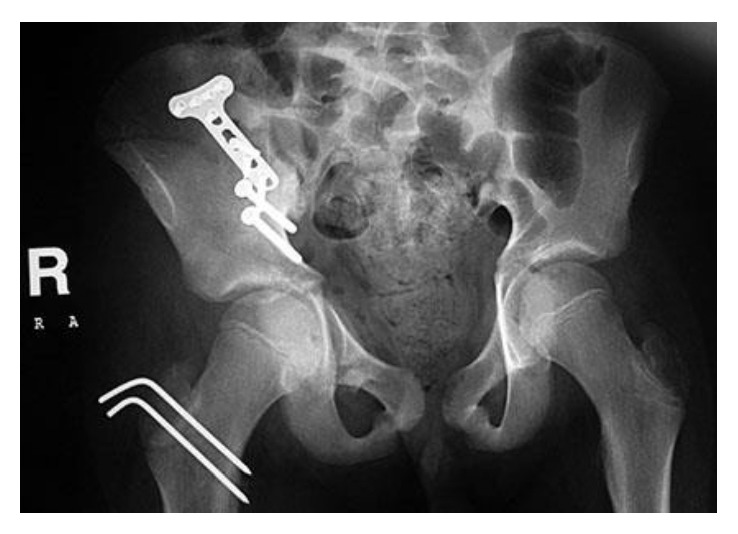
Postoperative AP pelvic X-ray showing adequate open reduction internal fixation.

Once in place, the anterior column fracture was reduced anatomically using a specific acetabular clamp which allowed maintaining the reduction while fixing using first two 4.5 mm anteroposterior cortical screws placed at the level of the pelvic brim. Then we added a 3.5 mm LCP T-plate which allowed reinforcing anterior column fixation. The quadrilateral surface was reduced and press-fitted to its anatomical place. The greater trochanter tuberosity was reduced anatomically and fixed with two Kirchner wires. Closure of the 2 incisions was done over 2 suction drains. Immediate post-op AP pelvis X-ray showed adequate reduction and fixation of the fracture dislocation (Figure 3).

Two days after the surgery, non-weight-bearing ambulation was initiated for a period of 3 months using a walker. Range of motion exercises was also initiated and increased progressively according to pain tolerance. The patient was discharged uneventfully from the hospital at day 6 post-op, the stitches were removed at day 14, and continuous physiotherapy was carried out on a daily basis. Monthly follow-up hip X-rays were obtained (M1, M2, and M3) which showed adequate healing, the quadrilateral surface holding properly in place.

At 3 months post-op, he was readmitted to the hospital for pin removal from the right greater tuberosity which was done on a one-day surgery basis. As tolerated weight bearing was started with rapid progression to full weight bearing over a month, the patient was pain-free with slight limitation in extreme range of motion compared to the contralateral hip. At 4 months post-op, he was able to ambulate with full weight bearing and no hand support but with some limitation in flexion and internal and external rotation.

**Figure 4:**
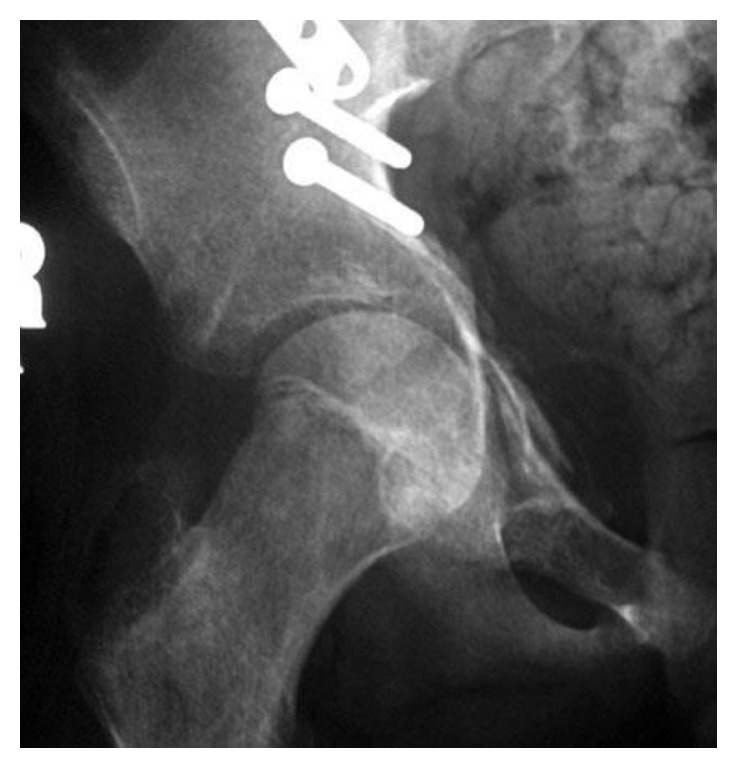
Medial displacement of both the quadrilateral surface and femoral head resulting in focal joint space narrowing at the superomedial joint surface.

At the 6-month follow-up visit, the patient was complaining of mild hip pain, and on physical examination, he was found to have decreased significantly his right hip range of motion with significant stiffness. Anteroposterior radiographs of the pelvis and lateral radiographs of the hip were obtained and showed a millimetric medial displacement of both the quadrilateral surface and femoral head resulting in focal joint space narrowing at the superomedial joint surface (Figure 4).

The patient was then seen every 2 months; each time the patient was examined, and radiographs were obtained. On physical examination, the patient was found to have a stiff hip with 20° of hip flexion only, the pain on weight bearing being very mild. The patient at this period of time had already finished all the previously prescribed physiotherapy sessions noting that they were of no benefit in the last few sessions.

**Figure 5:**
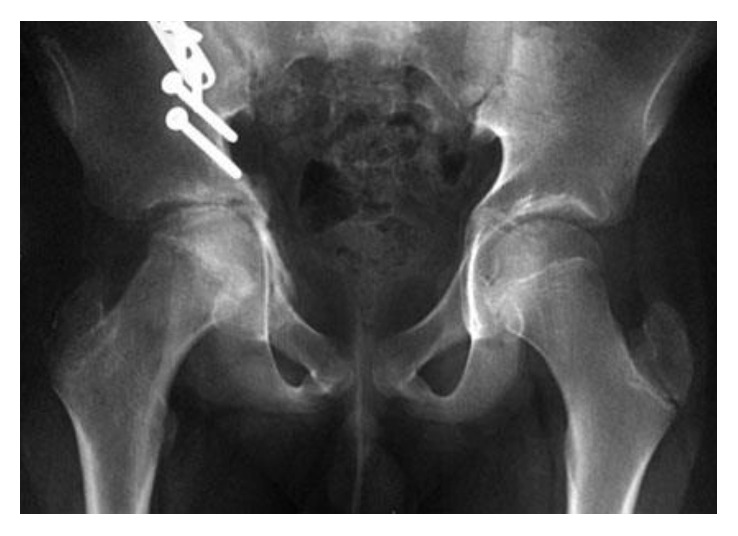
Terminal right hip osteoarthritis.

We did not feel that an early post-op MRI checking the head vascularity or the surface cartilage status would be of any use to our management plan. On follow-up radiographs, the patient was found to have progression of joint degeneration till reaching, after two years, a severely degenerated joint with joint space narrowing and femoral head sclerosis with possible avascular necrosis (Figure 5).

Due to the very young age of the patient, we still have not decided yet for additional treatment like a hip replacement arthroplasty.

## 3. Discussion

In pediatric acetabular fractures, the orthopedic surgeon is confronted with a unique pattern of injury. While reviewing the literature, we find pediatric pelvic fractures to be rare and acetabular fractures even rarer. The lack of experience with this type of fractures leads to unavailability of standardized protocols for therapy. In the course of treatment, we were confronted with two major issues. The first consisted of the entrapment of the femoral head with the subsequent delay in management, and the second was the nonfixation of the quadrilateral surface which might have led to progressive medial migration of the femoral head.

The mechanism of injury in our case is unique. We hypothesize that a direct lateral impact over the greater trochanteric region caused the femoral head to be pushed anteriorly and medially causing, in turn, a displaced anterior column fracture radiating to the iliac wing and the quadrilateral surface to fracture. The initial anterior displacement of the anterior column at the moment of the impact caused an enlargement of the anteroposterior diameter of the acetabulum allowing the femoral head to migrate medially fracturing the quadrilateral surface to the inner pelvis. The entrapment of the femoral head medially was caused by the fact that the initial displacement of the anterior column was partially spontaneously reduced by bone plasticity, thus decreasing the anteroposterior diameter of the acetabulum and squeezing over the femoral head-neck junction causing the central entrapment. This mechanism is paramount to understand because it is a source of delay in reduction and possible subsequent avascular necrosis. If the first surgeon was aware of this possible entrapment, he would have transferred the patient immediately to a level I trauma center where the staff is more prepared to undertake immediate open reduction internal fixation in case of failure of closed reduction.

The second issue was the lack in the market, at the time of surgery, of the adequate quadrilateral acetabular plate that might have held it in place. We thought intraoperatively that press fitting the quadrilateral surface to its anatomical location and keeping the patient non-weight bearing for 3 months post-op would be enough for the surface to heal and to prevent further medial displacement. We were in fact wrong, and progressive medial displacement have occurred causing edge loading of the femoral head over the inner rim of the acetabular cartilage. This mechanism triggered wearing away of the cartilage with progressive joint space narrowing and terminal hip osteoarthritis. On the other hand, the initial traumatic impact to the cartilage may lead to terminal hip osteoarthritis anyway.

We recommend therefore early conversion to open reduction internal fixation in cases of pediatric central fracture dislocation of the hip that fail closed reduction. Second, in cases of medial displacement, we recommend the use of modern plates that fix the quadrilateral surface, in order to prevent secondary displacement that might increase the likelihood of secondary osteoarthritis.
